# Wood-Based Micro-Biochars in a Cement Mixture

**DOI:** 10.3390/molecules30091898

**Published:** 2025-04-24

**Authors:** Minkyeong Pyo, Jongsun Kim, Seungwook Seok, Chan Ho Park, Wonchang Choi

**Affiliations:** 1 Department of Chemical, Biological and Battery Engineering, Gachon University, Seongnam 13120, Republic of Korea; minrudxx5174@gachon.ac.kr; 2 Department of Architectural Engineering, Gachon University, Seongnam 13120, Republic of Korea; kjswno1@gachon.ac.kr (J.K.); sseok@gachon.ac.kr (S.S.)

**Keywords:** biochar, wood, reinforcement, concrete, carbon sequestering

## Abstract

Immediate action is required to achieve carbon neutrality within the cement industry. The integration of biochar into cement as a component of reinforced concrete has potential to mitigate carbon emissions in the construction sector by enabling carbon sequestration. In pursuit of eco-friendly practices and improved physical properties of cement composites, this study investigated the properties of wood-based, micron-sized biochar as a non-carbonate raw material, including its chemical composition, morphology, and wettability. The characterization of lignocellulosic micro-biochar and its mechanical impact on cement composites was a focus of this study. Cement was partially replaced with varying weight percentages of micro-biochar (1, 3, and 5 wt%), and the effects were evaluated through compressive strength tests after 7 and 28 d. The results demonstrated that the micro-biochar could sustain strength even when substituted for cement. Notably, after 28 d, the compressive strength of the sample with only cement was 29.6 MPa, while the sample with 3 wt% biochar substitution showed 30.9 MPa, indicating a 4.4% increase. This research contributes to sustainable construction practices by offering a green solution for reducing carbon emissions in the industry.

## 1. Introduction

Cement production is notorious for its significant carbon footprint, accounting for a substantial portion of all global CO_2_ emissions. In particular, in 2021, cement production generated a staggering 2.6 billion tons of CO_2_ emissions, representing over 7% of all global carbon emissions, with emissions more than doubling since 2002 [1]. The high carbon intensity of cement is attributable to three main factors: direct emissions resulting from fuel combustion (40% of greenhouse gas (GHG) emissions), indirect emissions stemming from electricity usage and transportation (10%), and emissions from material consumption, including the decarbonization process of limestone as a primary raw material for cement (50%) [2]. Extensive research and analysis have been conducted to adopt low-carbon technologies, such as improving energy efficiency and recovering waste heat. However, it remains difficult to develop carbon reduction technologies that take into account all the omnidirectional carbon emission channels related to cement. In this regard, a sustainable solution is to reduce the quantity of cement raw materials used by seeking material substitution without deteriorating quality.

To tackle this critical challenge and reduce CO_2_ emissions, one approach is to use alternative non-carbonate raw materials that contain calcium oxide and magnesium oxide instead of limestone, and to increase the proportion of blending materials, which include recycled materials, as substitutes for the cement content. Such measures have the potential to reduce CO_2_ emissions significantly [3]. In Korea, for example, regulations (KS L 5201) permit the incorporation of blast furnace slag, pozzolan, fly ash, and limestone as admixtures for Portland cement production, albeit with restrictions on their types and proportions. The type and proportion of admixtures are limited to either blast furnace slag, pozzolan, or fly ash within 5%, with limestone powder comprising the remaining 5% [4].

To reduce CO_2_ emissions and enhance the physical properties of cement composites, this study investigated the potential benefits of using wood-based biochar as a non-carbonate raw material to substitute for cement. Biochar is a char that is obtained by the thermal decomposition of biomass in an oxygen-limited environment. Common biomass sources include woody material, food waste, agricultural waste, and industrial waste. The type of biomass determines material properties like the yield, physicochemical properties, porosity, and carbon content of the biochar produced, while production methods and conditions, including time and temperature, also determine the proportion of the substances produced [5]. Typical biochar production methods include pyrolysis (a treatment that removes lignin from cellulose), hydrothermal carbonization, gasification, torrefaction, and flash carbonization [6]. Depending on the pyrolysis temperature and rate, different proportions of biochar are produced. To achieve a higher yield of biochar, a lower pyrolysis temperature and slower pyrolysis rate are adopted [7]. As the temperature increases, the proportions of nitrogen [8], oxygen, and hydrogen decrease, and the atomic content of carbon increases [9]. However, the carbon recovery rate declines when the temperature exceeds 550 °C. Therefore, the production temperature of biochars for the purposes of this study ranges from 450–550 °C. This temperature range is optimal for achieving high-yield production and moderate carbon content in biochars, while minimizing residual organics. According to Qambrani et al. [10], the production method that uses slow pyrolysis (0.6–120 °C min^−1^) and hydrothermal carbonization achieved the highest yield (88.9%) of wood-based biochar, with a carbon content of more than 80%. Although biochar has been primarily used as a soil amendment or carbon sequestration material, it also holds promise for advancing the use of biochar–cement composites in concrete structures.

Limited studies in the literature have examined the utilization of biochar in cement composites, though existing research showcases its diverse applications and beneficial effects. Choi et al. [11] explored the incorporation of biochar obtained from slow pyrolysis, emphasizing its carbon sequestration potential, as a partial substitution (0–20 wt%) for cement in cement composites. Gupta et al. [12] conducted a study focused on evaluating cement mortar by incorporating 1–2% biochar. Compared to conventional cement mortar, compressive strength was enhanced and permeability was reduced. These results suggest that the biochar in the mortar acted as a micro-filler, thereby promoting cement hydration. Furthermore, it was reported that biochar could adsorb up to 1.67 mmol of CO_2_ per 1 g, indicating its potential to serve as a carbon-sequestering material in concrete constructions. Han and Choi [13] contributed insights into the material properties of cement composites containing wood-based biochar, revealing a notable 12% increase in the compressive strength of cement mortar when augmented with wood-based biochar as opposed to conventional cement mortar. Javed et al. [14] experimentally investigated the effects of various biochars on the physical, mechanical, and microstructural properties of cement pastes and mortars. Their findings confirmed that the addition of biochar resulted in the formation of more hydration products with 2 wt% doses of biochar, and the biochar was found to adsorp 1.25–1.72 mmol g^−1^ of CO_2_. Sirico et al. [15] confirmed the effect of biochar extracted from forestry waste residues on the physical and mechanical performance of concrete by adding biochar up to 5% of the cement weight during the mixing process. In particular, after long-term dry curing over 1 yr, the moisture retained within the pores of biochar was gradually released, which facilitated the hydration of cement and consequently enhanced its strength.

In a distinctive approach, Suarez-Riera et al. [16] utilized biochar as nano/microparticles in optimized proportions within cement composites. Their mechanical properties testing demonstrated heightened strength and toughness in the resulting cement composite specimens, without losing ductility. Eom et al. [17] focused on flexural strength characteristics by investigating mortar with partial substitution of aggregate (up to 9.3%) by biochar, while maintaining a constant cement ratio. Their findings underscored the significant impact of the biochar blend ratio on the flexural strength of the mortar, resulting in an improvement of up to 20.68%.

Ali et al. [18] conducted a comprehensive examination of biochar derived from sawdust, employing advanced techniques such as field emission scanning electron microscopy (SEM), X-ray diffraction (XRD), thermogravimetric analysis (TGA), and isothermal calorimetry. Their research specifically delved into the compressive strength of cementitious composites by replacing cement paste samples with 1, 3, and 5 wt% of biochar. The results confirmed that the inclusion of biochar contributes to the enhancement of cementitious compounds, specifically in terms of improved hydration products. Together, these studies underscored the diverse applications and positive effects of biochar in augmenting various properties of cement composites.

Due to its inherent porosity, the content of biochar contributes to superior water retention capacity within cement composite materials [11]. Consequently, researchers have investigated the porous structure of biochar to reduce its density and enhance its permeability as the quantity of biochar incorporated into the mixture is increased. In this study, the components of wood-based biochar were identified using particle size distribution (PSD), SEM/energy dispersive X-ray (EDX) spectrometry, Fourier transform infrared (FTIR) spectrometry, XRD, and TGA. The strength-enhancing effects and carbon emission impacts of cement composites were then evaluated based on the inclusion rate of the wood-based biochar.

In summary, prior research by Choi et al. [11], Gupta et al. [12], Han and Choi [13], and Suarez-Riera et al. [16] has consistently demonstrated the positive impact of incorporating biochar into cement mortar and composites. This impact is particularly notable in terms of enhancing compressive strength, toughness, and overall mechanical properties. Additionally, the porous structure of biochar has been identified as beneficial for improving permeability [19] and reducing density [20] in cement composites. Nevertheless, a more thorough investigation is essential to comprehend the size-dependent chemical and physical properties of wood-based biochar. Characterizing its particle size distribution, density, permeability, and microstructural characteristics using techniques such as SEM, PSD, BET, SEM/EDX, FTIR spectrometry, and XRD analysis can provide valuable insights into these properties. Therefore, the objective of this study is to identify and assess the physical properties of wood-based biochar based on its substitution rate in cement composite, examining its effects on strength. Through a systematic exploration of various inclusion ratios of wood-based biochar, this study aims to offer comprehensive insights into its potential for enhancing the physical performance of cement-based materials. The findings of this study are anticipated to contribute to the advancement of environmentally friendly practices in the cement industry, fostering the development of more sustainable construction materials and promoting a greener future.

## 2. Experimental Section

### 2.1. Materials

Biochar flake (hardwood-based), purchased from Charcoal House (Crawford, NE, USA), was obtained via 450 ± 100 °C pyrolysis. Super P (conductive carbon black) was purchased from MTI Korea (Seoul, Republic of Korea). Graphite (<20 μm) and other samples were purchased from Sigma–Aldrich (St. Louis, MI, USA). Cement was prepared according to KS L 5201 [4], and sand was prepared according to KS L ISO 679 [21].

### 2.2. Fabrication of μ-Biochar

Micron-sized biochar (μ-biochar) was obtained by grinding biochar flake using a roll-mill (Lab Roll Mill, TENCAN, Changsha, China). The roll-mill was operated with 100 g of biochar flake at 250–300 rpm in both forward and reverse directions for 15 min each, for a total of 30 min.

### 2.3. Moisture Absorption of Biochar

Initially, we measured the weight of the biochar flake and prepared an equivalent weight of μ-biochar for comparison. We placed each biochar sample, with different particle sizes, into individual vials, and dried them in a vacuum oven at 100 °C under negative pressure conditions to remove the absorbed moisture of samples. After 24 h, the weight of the completely dried biochar samples was measured as an initial weight. Then, the samples were exposed under the saturated moisture condition (relative humidity, RH, 100%), and the change in the weight of the samples was periodically monitored.

In our experiment, the biochar sample was dried under specified conditions and then exposed to water absorption in a defined environment. As a result, according to ASTM D2654-22 [22], we calculated the outcomes using the moisture regain formula:

$$Moisture\;Regain\;(\%)=\frac{100(A-D)}{D}$$

where:

A—mass of biochar in moisture-equilibrium at specified conditions;
D—mass of dried biochar.

### 2.4. Compressive Strength Test of Biochar and Cement Mortar Mixture

The materials were mixed at 200 ± 10 rpm using a mixer according to the KS L 5109 [23]. Initially, μ-biochar, cement, and sand were combined and mixed for 1 min. Water was then introduced, and the mixture was blended for an additional 3 min. After casting, the specimens were cured in a humid environment for 24 h and then submerged in a curing tank at 20 °C for 7 and 28 d.

### 2.5. Characterization

The structure and morphology of samples were examined using SEM (SU8600, Hitachi (Tokyo, Japan), and VEGA3 SBH, TESCAN (Brno, Czech Republic)), and the size of biochar flake was obtained. The size of μ-biochar was obtained by PSD (LS 13 320, BECKMAN COULTER, Tokyo, Japan) analysis, within a measurement range of 0.375–2000 μm, employing a laser diffraction and scattering method. The pore structure of the biochar was analyzed using BET and BJH methods (TristarⅡ 3020, Micrometrics, Norcross, GA, USA) based on nitrogen gas adsorption at 77K. The surface area of the biochar was analyzed via the BET method, and the pore size and volume distribution was analyzed via the BJH method, determined from the point at P/P_0_ = 0.994. The elemental compositions of the biochar were analyzed using SEM/EDX (SU8600, Hitachi, Tokyo, Japan) spectroscopy. FTIR (Vertex 70, Bruker, Billerica, MA, USA) spectra of all samples were obtained in the range of 3600–700 cm^−1^, and XRD patterns of the biochar were obtained using an XRD (EMPYREAN, Panalytical, Almelo, The Netherland) with 2θ = 10–90° to analyze the structural properties. To check the moisture regain in biochar, TGA (SDT Q600, Ta instruments, New Castle, DE, USA) was performed under nitrogen conditions with a heating rate of 10 °C min^−1^. UTM (HCT-CH 100, Heungjin, Gimpo, Republic of Korea) was used to measure the strength of cement mortar containing biochar, using a 50-ton capacity UTM until the specimens failed. In accordance with ASTM C109 [24], cube-shaped specimens with dimensions of 50 mm × 50 mm × 50 mm were used.

## 3. Properties of Wood-Based Biochar

### 3.1. Structural Characterization of the μ-Biochar

The biochar used in this study was obtained via 450 ± 100 °C pyrolysis of mixed hardwood. Note that, in the case of woody biochar, the produced solid particles are large and, in order to incorporate woody biochar into cement composites, the biochar must be ground to a particle size similar to that of cement. Therefore, considering that cement typically has a particle size of 10–50 μm, and Gupta et al. [25] found that biochar particles range in size from 2–100 μm, the samples must be ground into a powder. For this purpose, we first ground the wood-based biochar at 250 rpm for 30 min using a roll-mill, and then used the resultant sample for composition analysis. We analyzed the resultant woody biochar using SEM, PSD, Brunauer–Emmett–Teller (BET), and SEM/EDX analysis.

**Figure 1:**
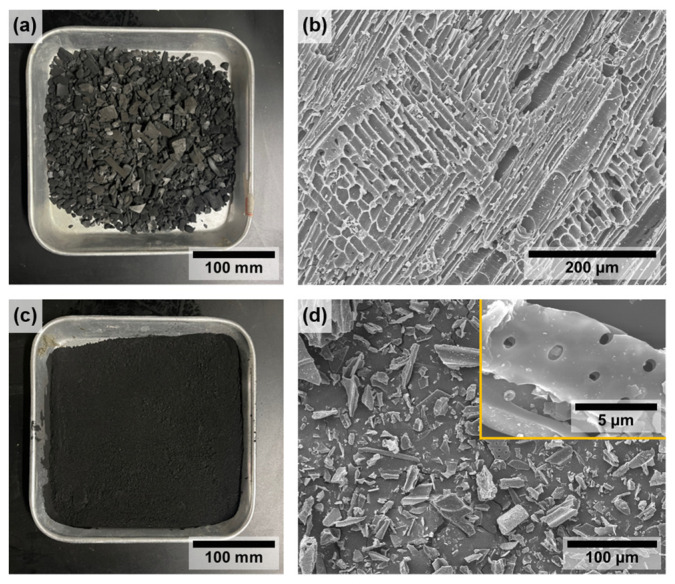
(**a**) Photograph and (**b**) SEM image of biochar flake, and (**c**) photograph and (**d**) SEM image of μ-biochar.

Figure 1 shows digital photographs and SEM images of the wood-based biochar for the flake and micro powder types. The commercial wood-based biochar exhibited a diverse range of particle sizes. We visually confirmed that the biochar was ground to a smaller size (Figure 1a,c). As a result of observing the surface through SEM images as shown in Figure 1b, the surface morphologies of biochar flakes were a complex network of pores, channels, and honeycomb shapes together with a fibrous surface [26] formed by wood fibers including xylem and phloem structures [27]. The dimensions and aspect ratio of the pores were determined to be 13.8 μm and 2.3, respectively, which aligns with the findings of previous literature (Figure S1 and Table S1).

In contrast, the wood structures were approximated in the μ-biochar particles following the grinding process (Figure 1d). The presence of small pores is still evident, as shown in the high-magnification view in Figure 1d, which depicts an average size of 1.4 μm and an aspect ratio of 1.4 (Figure S2 and Table S2). The carbonized structures observed in these wood-based biochars are distinct from those derived from other biomass at temperatures exceeding 500 °C, which exhibit disordered and cracked surfaces [28,29,30].

**Figure 2:**
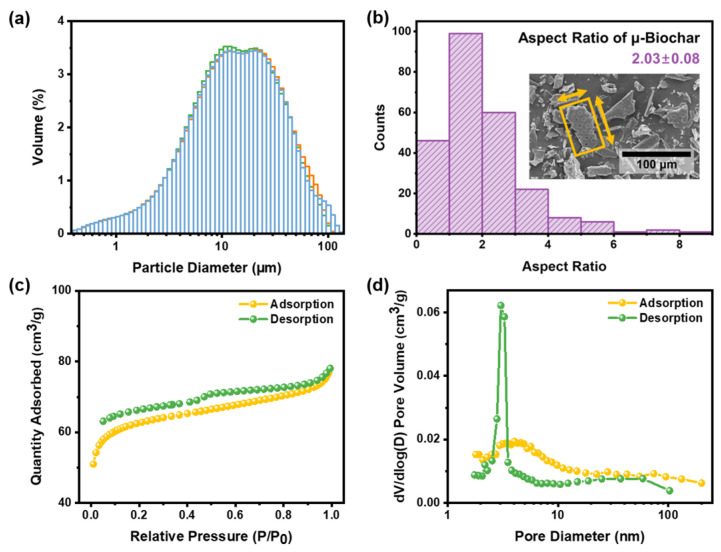
(**a**) Particle size distributions of μ-biochar from triplicate measurements, are shown in blue, green, and orange. (**b**) Histogram of the aspect ratio of μ-biochar, calculated using the minimum bounding rectangle highlighted in yellow. Arrows indicate the corresponding long and short axes. (**c**) N_2_ adsorption–desorption isotherm of μ-biochar. (**d**) Pore size distribution calculated using the BJH method.

We characterized the distribution of size and porosity of μ-biochar, as shown in Figure 2. The wood-based biochars were ground using the roll-milling process to produce μ-biochar by reducing the average size. The degree of size reduction was adjusted by the revolutions per minute (rpm) and the milling duration. The sample designations D10, D50, and D90 indicate that 10, 50, and 90% of the particles in the respective samples are smaller. Figure 2a illustrates the PSD of the μ-biochar, and Table S3 shows the relevant triplicated information for the D10, D50, and D90 samples, with average values as follows: 3.47 μm for D10, 14.21 μm for D50, and 46.64 μm for D90, resulting in an average particle size of 20.65 μm. Notably, over 90% of the samples exhibit sizes smaller than 100 μm. According to Gupta et al. [31], biochar with a particle size of 20 μm or smaller is anticipated to impart filler and pore-blocking effects. The result of particle size of the μ-biochar indicates that the μ-biochar is in the proper size range to incorporate into a cement composite mixture, significantly influencing the composite’s performance. An additional benefit of the grinding process is the reduction in particle size and exposure of abundant micropores, which together significantly increase the surface area of the biochar, leading to excellent water absorption and retention capacity. These characteristics allow the pulverized biochar to act as a hygroscopic filler in cement composites, which inherently lack such water-handling ability. This highlights the multifaceted influence of biochar particle size on the physical properties and behavior of the composite material [32]. Figure 2b depicts a histogram illustrating the aspect ratio of the biochar particles derived from the SEM image. The aspect ratio was calculated by measuring the major and minor axes, with the assumption that each particle could be approximated as a rectangle using image analysis software (Image J, ver. 1.53e). The analysis revealed that the mean aspect ratio of the biochar particles was 1:2.03.

To gain a deeper understanding of the porosity and free volume of the μ-biochar, we performed N_2_ adsorption/desorption analysis. According to the IUPAC classification, the isotherm plot shown in Figure 2c is identified as Type IV, accompanied by a hysteresis loop (H4). Type IV isotherms are characteristic of mesoporous structures, and H4 hysteresis loops are observed in micro-mesoporous carbon. These features indicate that the wood-based biochar is a micro/mesoporous carbon material with predominantly mesoporous characteristics [33]. The specific surface area was determined to be 244 m^2^ g^−1^ using the Brunauer–Emmett–Teller (BET) method, which aligns with values reported in a previous study (150–250 m^2^ g^−1^) [26]. At a relative pressure of P/P_0_, the total pore volume of pores smaller than 314.932 nm in diameter was calculated to be 0.12 cm^2^ g^−1^. In contrast, the Barrett–Joyner–Halenda (BJH) method accounts only for pores within the 1.7–300 nm range, yielding total pore volumes of 0.024 and 0.020 cm^3^ g^−1^ for adsorption and desorption, respectively, as shown in Figure 2d. These values are also similar to the biochars pyrolyzed under high temperatures over 500 °C (0.0209–0.0317 cm^3^ g^−1^) [34]. Additional pore characteristics are summarized in Table S4, and the portion of the micro/mesopores (up to 50 nm) of the total surface area was over 90% (up to 85% mesopores), as shown in Figure S3. Following the rigorous pyrolysis and pulverization process, the cellular structure-driven fibrous morphology was partially preserved, and porous features were generated through decomposition. This resulted in the formation of micro- and mesopores and a relatively larger specific surface area than that observed in other carbonized organics. Lehmann [35] and Shafie et al. [36] found that water retention is maximized when the pore size is 30 μm or smaller. In other words, these micro/mesopores exhibit the capacity to retain and release water during the hydration reaction in the cement composite, suggesting a potential enhancement in strength [25].

### 3.2. Elemental Analysis of the μ-Biochar

**Figure 3:**
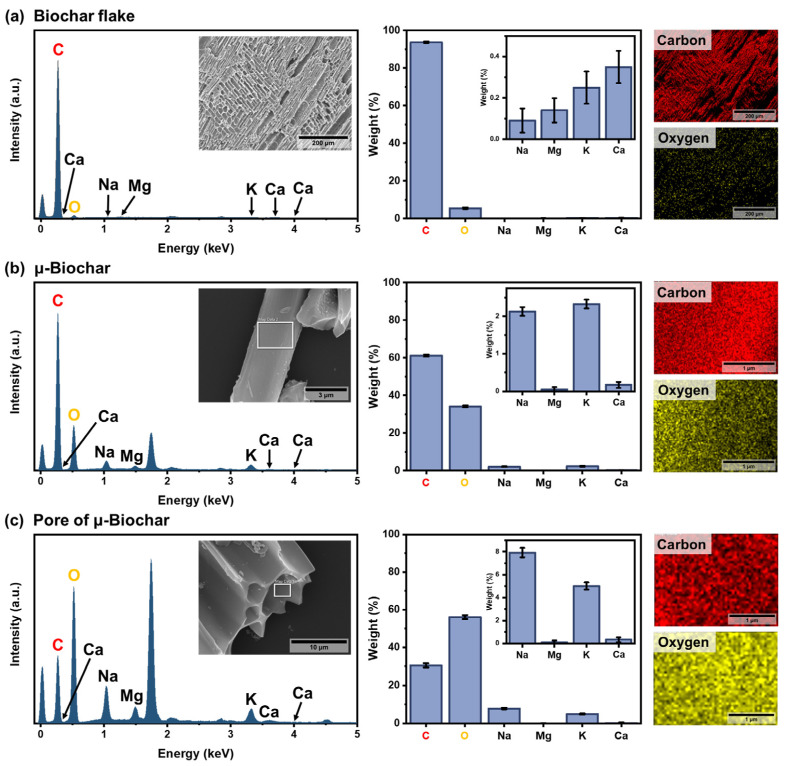
SEM–EDX analysis of (**a**) the surface of a biochar flake, (**b**) the surface of μ-biochar, and (**c**) the inner surface of a μ-biochar pore. Elemental spectra and quantification (with 95% confidence intervals) were obtained from the white-framed regions in (**a**–**c**). Corresponding elemental maps show the distribution of carbon (red) and oxygen (yellow).

SEM/EDX analysis was employed for elemental analysis to explore the chemical composition of the microstructure, as shown in Figure 3 and Figure S4. In the SEM/EDX spectrometry graph, the primary crystalline phases identified include carbon, oxygen, sodium, magnesium, potassium, and calcium. Table 1 quantifies the averages and the 95% confidence interval results of SEM/EDX analysis, and the numerical data are presented in Table S5. Carbon emerges as the predominant crystal phase, closely followed by oxygen in the case of biochar flake (93.70 ± 0.41% carbon and 5.48 ± 0.39% oxygen). The elemental composition of the wood-based biochar flake in our study aligns with findings by Gupta et al. [37], where woody biochar exhibited 87.13% carbon and 7.21% oxygen. This consistency suggests a similarity in the composition of the biochar used in our study, and the carbon content of wood-based biochar appears relatively higher than other biomass-based biochars, as shown in Table 1. According to research by Spokas [38], a semi-permanently chemically stable biochar is characterized by an oxygen-to-carbon (O/C) ratio of less than 0.2. Our wood-based biochar flake demonstrates an O/C ratio of 0.06, indicating its potential for chemical stability. Moreover, the results of SEM/EDX spectrometry reveal clear differences in elemental distribution depending on the particle size and the specific observed point on the biochar. Compared to the biochar flake, the μ-biochar contains approximately six times more oxygen (O/C ratio = 0.58) on its surface. The inner surface of the pore channel of μ-biochar contains about 1.6 times more oxygen than the surface (O/C ratio = 1.83). This indicates that the oxygen-containing moieties and water molecules might be more readily absorbed by μ-Biochar than by biochar flake. This can be attributed to the hydration reactivity of μ-biochar in the cement composite.

**Figure 4:**
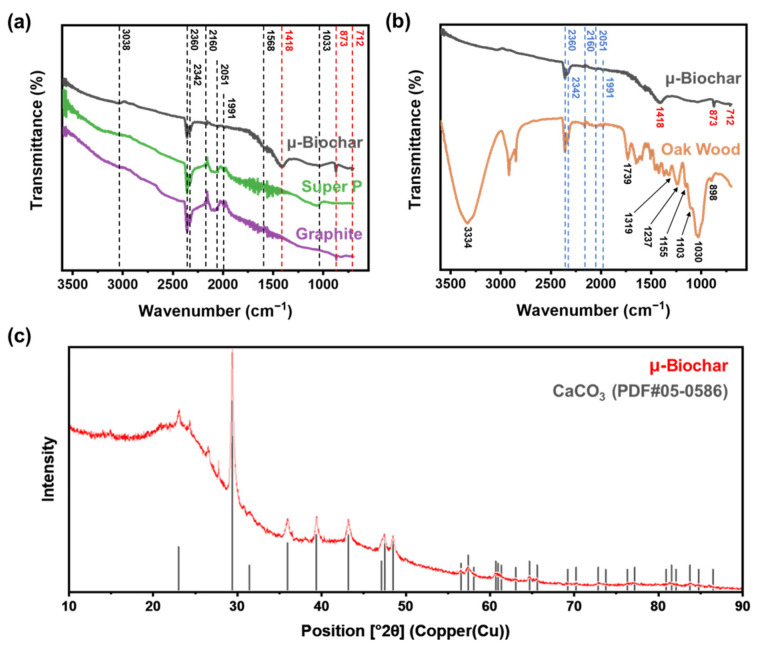
(**a**) ATR-FTIR spectrum comparing μ-biochar with Super P and graphite; (**b**) ATR-FTIR spectrum comparing μ-Biochar with oak wood; and (**c**) X-ray diffraction pattern of μ-biochar.

The μ-biochar was characterized using attenuated total-reflectance Fourier transform infrared (ATR-FTIR). In Figure 4a, the ATR-FTIR spectrum shows bands at 3038, 2051, and 1991 cm^−1^ for C-H stretching vibration, bands at 2360 and 2342 cm^−1^ for CO_2_, a band at 2160 cm^−1^ for C≡C, and bands at 1568 and 1033 cm^−1^ for C-O. The distinctive peaks were also observed in analogous carbon materials, including Super P (a type of carbon black) and graphite samples. This suggests that μ-biochar exhibits chemical properties that are nearly identical to those of general carbon materials. The sole distinction point was the appearance of distinctive peaks at 1416, 873, and 712 cm^−1^ in μ-biochar, indicative of the presence of CaCO_3_. A comparison of the ATR-FTIR peaks between the initial (oak wood) and final (μ-biochar) stages of biomass pyrolysis is presented in Figure 4b. The oak wood spectrum exhibits the following characteristics: 3334 cm^−1^ for OH stretching vibration of cellulose, 1733 cm^−1^ for C=O stretching vibration in cellulose and hemicellulose, and 1319, 1237, 1155, 1103, 1030, and 898 cm^−1^ for C-O stretching vibration. The intensity of these peaks either diminished or disappeared in the biochar spectrum, indicating that as oak wood underwent pyrolysis to form biochar, the OH and CO chemical compounds underwent carbonization and subsequently disappeared. Figure 4c illustrates the XRD results, which corroborate the crystalline structure of the material. The μ-biochar displays a heightened peak at 2θ, spanning 29–31°, and no additional specific peaks, attributable to its amorphous nature. This finding aligns with observations documented in previous studies [49,50]. Furthermore, as observed in the FTIR data, peaks corresponding to limestone (CaCO_3_) were identified in the XRD spectrum. The raw material of the μ-biochar is wood, which contains calcium carbonate as part of its intrinsic composition. Therefore, calcium components were detected in wood-based biochar even after pyrolysis. In this regard, the XRD patterns observed in our study were similar to those reported for other woody material-based biochars, including oak wood and soybean stover [51].

**Figure 5:**
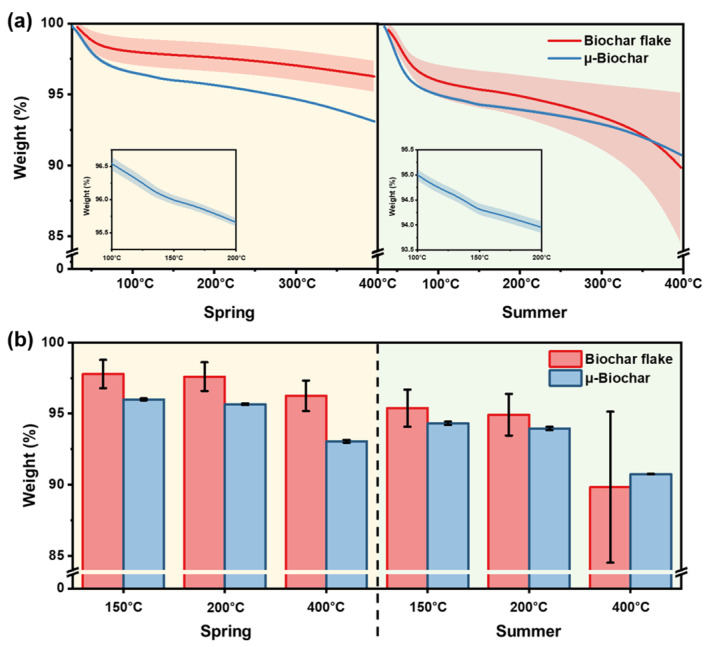
(**a**) TGA profiles of biochar flake and μ-biochar measured during the spring (10.1 °C, RH: 54.5%) and summer (29.9 °C, RH: 74.8%) seasons. Shaded areas represent standard errors from triplicate measurements. (**b**) Residual weight (%) of biochars at 150, 200, and 400 °C.

In Figure 5, the exact moisture content of biochar was determined by temperature using TGA, and the differences between samples were assessed based on the type of biochar (biochar flakes and µ-biochar). In this experiment, the moisture content of biochar was classified and compared by season, reflecting the environmental conditions under which a significant amount of biochar is stored before it is used as a cement composite in an industrial environment (Table S6). As illustrated in Figure 5a, the data demonstrate that µ-biochar exhibits greater weight loss than biochar flake, with its relatively larger particle size. Most of the weight loss occurred rapidly up to approximately 100 °C, due to the presence of moisture impregnated within the biochar. The discrepancy in moisture content attributable to the differing sizes of the biochar samples was more pronounced during the spring season than it was during the summer. Additionally, Figure 5b illustrates that µ-biochar exhibits a markedly reduced error range of TGA results in comparison to biochar flake. This indicates that the discrepancy in moisture content between samples is minimal in µ-biochar, which offers an advantage in terms of process reliability and consistency in the properties of cement composite. It is notable that weight loss at temperatures exceeding 300 °C was detected in both biochar flakes and µ-biochar. This phenomenon can be attributed to the additional degradation of residual organic matter in the biochars. Therefore, it is reasonable to compare the moisture content of the biochar samples based on the results obtained at temperatures below 300 °C.

**Figure 6:**
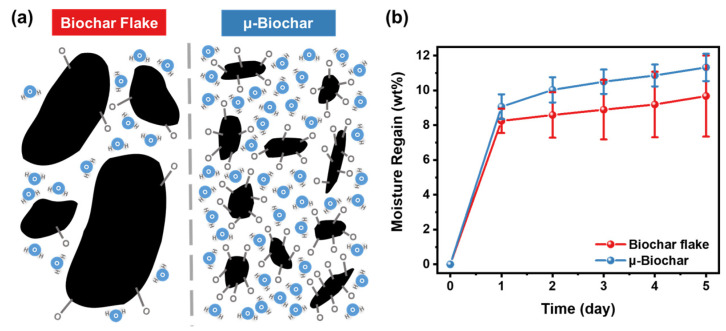
(**a**) Scheme of moisture absorption behavior, and (**b**) comparison of moisture regain of biochar flake and µ-biochar with 95% confidence intervals.

We conducted the moisture absorption experiment according to ASTM D2654-22 [22]. This method is used to confirm the water absorption characteristics of biochar in a saturated vapor, while the TGA is used to assess the intrinsic moisture content of the as-synthesized biochar. This results in smaller particles being more effective at moisture retention, as shown in Figure 6b. Consistent with the results in Figure 5, µ-biochar retains more moisture and shows minimal variation sample-by-sample, as compared to the biochar flake (Figure S5, Tables S7 and S8). Based on the data from the fifth day in storage, the µ-biochar absorbed 11.3 ± 0.8 wt% moisture, while the biochar flake absorbed 9.7 ± 2.3 wt%. As shown in Figure 6a, the biochar flake has fewer exposed oxygen-containing groups on its surface due to its limited porosity. In contrast, the µ-biochar has a high O/C ratio on the surface and a relatively uniform specific surface area. As a result, the biochar flake has a lower moisture content than the µ-biochar, and the µ-biochar maintains a more consistent moisture content within a storage environment. This suggests that the µ-biochar is a safer choice for concrete applications in terms of reliable processing.

## 4. Properties of Cement Mortar Containing μ-Biochar

We prepared four cement mortar mixtures that contained µ-biochar for mechanical characterization. Table 2 shows the batch mixture proportions for this cement mortar, with the biochar content varying from 0–5%, designated as BC_0%, BC_1%, BC_3%, and BC_5%, respectively. The sand-to-cement ratio (s/c) was 2.75, and the water-to-cement ratio (w/c) was 0.485, in accordance with ASTM C109 [24].

Cement mortar flow tests, conducted in accordance with ASTM C1437 [52], are summarized in Table 3, which outlines the flow rates for each tested variable. Notably, as the content of µ-biochar increased, the flow value for the cement mortar consistently decreased. For the plain mortar (BC_0%), the flow value is recorded at 124.3 mm, surpassing those of the cement mortar formulations incorporating µ-biochar. Specifically, when 5% µ-biochar was introduced into the mortar, the flow value was reduced by 9.9% compared to the plain mortar. This finding aligns with prior research by Choi et al. [11], Gupta et al. [12], and Maljaee et al. [53], all of which observed that the high carbon content and porous structure of wood-based biochar lead to increased water retention. This water retention, in turn, contributes to a decrease in flow values correlating with the mortar’s workability.

**Figure 7:**
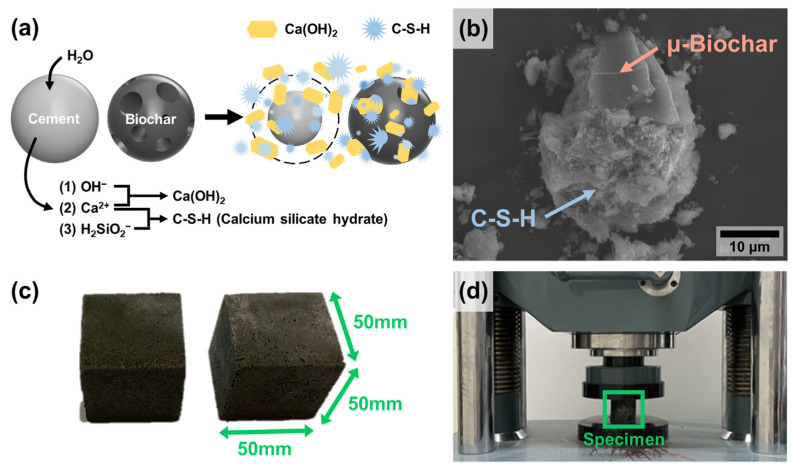
(**a**) Scheme of cement hydration process containing biochar, (**b**) SEM image of cement mortar containing biochar (BC), and photographs of the (**c**) BC_1% specimen and (**d**) compressive strength test setup.

In Figure 7, we replaced a certain amount of cement with mixed biochar to produce cement mortar. During the hydration reaction between cement and water, calcium silicate hydrate (C-S-H) and calcium hydroxide (Ca(OH)_2_) are formed, and this mechanism induces high strength in cement (Figure 7a). Figure 7b shows the microscopic morphology of cement mortar containing µ-biochar after the hydration process. Compared to the μ-biochar in Figure 1 and Figure 2, it is confirmed that hydrates such as C-H-S are generated and bonded to both the outer surface and within the pore channels of the biochar. This indicates that the biochar itself is a stable participant in the hydration reaction within the cement mortar. In other words, the µ-biochar influences the formation of hydration products, potentially leading to changes in the microstructure and mechanical properties of the cement mortar for the following reasons: (i) incorporating biochar into the cement composite results in the formation of numerous mesopores, and (ii) the mesopores of biochar exhibit the capacity to retain and release water during the hydration reaction, suggesting a potential enhancement in strength.

The compressive strength of biochar was tested in accordance with ASTM C109 [24] using cube-shaped specimens measuring 50 mm × 50 mm × 50 mm, as shown in Figure 7c,d. The compressive strength of concrete is the most important parameter in minimizing structural failures and ensuring the quality of buildings. The curing process of concrete typically requires 7 d to assess the initial development of strength and 28 d to reach full design strength. Thus, we measured the compressive strength of the cement mortar at 7 and 28 d in this study.

**Figure 8:**
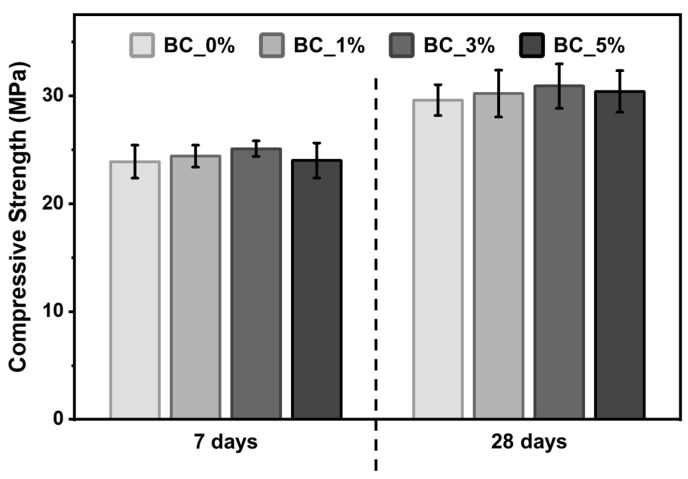
Compressive strength of cement mixtures containing µ-biochar after 7 and 28 d of curing.

The results of the compressive strength tests for the µ-biochar-containing cement mixtures are summarized in Figure 8 and Table S9. The compressive strength of the BC_0% sample was 23.9 ± 1.5 MPa at 7 d and 29.6 ± 1.4 MPa at 28 d. In comparison, the compressive strength of cement containing µ-biochar increased by 2.1, 5.0, and 0.4% at 7 d as the content increased to 1, 3, and 5 wt%, respectively. At 28 d, BC_0% was 29.6 MPa, an increase of 23.8% over the strength at 7 d. For the 1, 3, and 5% samples, compressive strength was increased by 2.0, 4.4, and 2.7%, respectively, compared to BC_0% at 28 d. The highest compressive strength was observed in the BC_3% specimen at both 7 and 28 d of curing. Furthermore, the compressive strength of the BC_5% specimen was found to be comparable to that of the BC_0% specimen. The increase in compressive strength with higher content of µ-biochar is attributed to the absorption and retention of free water within the cement matrix due to the porous structure of the biochar, resulting in higher compressive strength compared to BC_0% specimens [11,12]. Therefore, it is concluded that a 3 wt% substitution rate of biochar enhances compressive strength by approximately 4.4%, and up to 5 wt% substitution does not reduce compressive performance, indicating that biochar can partially replace cement.

This is consistent with the findings of Ali et al. and Lin et al., whose studies also reported that a 3 wt% substitution resulted in the highest compressive strength [18,19]. Choi et al. showed that replacing 5% of cement with biochar led to a 13.5% decrease in flowability and up to a 7% increase in compressive strength [11]. Javed et al. reported that, depending on the type of biochar, compressive strength increased by up to 13% within a substitution rate of 4 wt% [14]. Sirico et al. observed that compressive strength improved with curing time at substitution rates within 5 wt%, and after two years of curing, the strength was 26% higher than that of conventional concrete [15]. Since compressive strength varies significantly depending on the type of biochar and the length of the curing period, further comparative studies are needed.

## 5. Conclusions

This study systematically evaluated the structural, compositional, and mechanical characteristics of wood-based, micron-sized biochar to assess its applicability in cement composites. The utilization of biochar as a substitute for cement hinges upon the roll-milling-based grinding process and the reduction of particle size. Composition analysis showed a high carbon content (94%) and a low O/C ratio (0.06) in the biochar flakes, while µ-biochar showed a higher O/C ratio (0.58) and an average particle size of 20 µm. BET analysis confirmed its porous structure, which influences surface properties and workability in cement composites. Incorporating µ-biochar into cement composites improved compressive strength by 2.0–4.4%, reaching up to 30.9 ± 2.1 MPa after 28 d of curing. This enhancement is attributed to the filler effect and the internal curing behavior enabled by water absorption. These findings confirm that micron-sized, wood-based biochar can serve as a viable partial cement substitute.

## 6. Future Outlook

This study focused on the mechanical performance of biochar–cement composites. Based on these findings, future biochar utilization in the construction field should be considered in terms of its environmental and economic implications. Life cycle assessment (LCA) can provide valuable insights into the environmental footprint of biochar production and its integration into cementitious materials to achieve carbon sequestration. Given its CO₂ adsorption capability and origin from renewable biomass waste, biochar holds promise as a carbon-sequestering admixture to reduce the carbon footprint of cement-based materials. Techno-economic assessments are also essential to evaluate its cost-effectiveness and scalability for large-scale applications. Since biochar can be produced from discarded biomass, it offers a cost advantage over synthetic chemicals. Future studies should also explore diverse biomass sources and assess long-term durability, while expanding the utilization of biochar in multifunctional construction materials for sustainable, low-carbon development.

## Figures and Tables

**Table 1:** Elemental composition of several biochars.

Raw Material of Biochar	Elemental Composition (wt%)					
	Carbon	Oxygen	Sodium	Magnesium	Potassium	Calcium
Wood-based biochar flake [This work]	93.70	5.48	0.09	0.14	0.25	0.35
Wood-based μ-biochar [This work]	61.21	34.11	2.13	0.05	2.33	0.17
Inner surface of wood-based μ-biochar pore [This work]	30.57	56.08	7.93	0.08	5.01	0.32
Wood [37]	87.13	7.21	-	0.51	0.42	0.65
Food waste [37]	70.90	8.42	0.58	0.14	3.73	2.69
Rice husk [37]	66.22	13.63	1.98	3.40	2.69	0.11
Forest residues [39]	60	20	0.1	0.4	1.5	2
Agricultural waste [40]	67.5	25.3	0.2	0.7	2.1	2.5
Chinese biomass waste [41]	72.3	18.5	0.3	-	2.8	3.1
Orange peel [42]	65.1	21.7	-	0.3	1.9	1.7
Wood [43]	70	22	0.1	0.5	2	2.2
Rice husk [44]	55.6	34.1	0.3	-	3.4	1.8
Corn stover [45]	63.4	25.7	0.2	0.2	2.5	2.3
Bamboo [46]	75.3	18.9	0.4	-	2.3	3.4
Sawdust [47]	58.9	32.6	0.2	0.1	3.1	2
Coconut shell [48]	76.8	14.2	0.1	0.6	1.4	2.7

**Table 2:** Mixture properties of cement mortar with µ-biochar.

Specimen	Cement (g)	μ-Biochar (g)	Sand (g)	Water (mL)
**BC_0%**	1000	-	2750	485
**BC_1%**	990	10		
**BC_3%**	970	30		
**BC_5%**	950	50		

**Table 3:** Mortar flow test results.

Specimen	Mortar Diameter	Difference
**BC_0%**	124.3 mm	-
**BC_1%**	119.7 mm	−3.7%
**BC_3%**	116.3 mm	−6.4%
**BC_5%**	112.0 mm	−9.9%

## Data Availability

The raw data supporting the conclusions of this article will be made available by the authors on request.

## Supplementary Materials

The following supporting information can be downloaded at: https://www.mdpi.com/article/10.3390/molecules30091898/s1, Figure S1. SEM image of cells which demonstrates honeycomb structure of biochar flake. Figure S2. SEM image of pore of μ-Biochar. Figure S3. Ratio of micropore, mesopore, and macropore of μ-Biochar. Figure S4. SEM/EDX layered image and atom distribution of sodium, magnesium, potassium, and calcium of (a) surface of biochar flake, (b) surface of μ-Biochar, and (c) inner surface of μ-Biochar pore. Figure S5. Plots of triplicated moisture absorption test. Figure S6. SEM images of (a) biochar without mixing with cement, and (b–d) mortar containing biochar (BC). Table S1. Average size of cells of biochar flake. Table S2. Average size of pores of μ-Biochar. Table S3. Particle size distribution results. Table S4. Pore characteristics analysis of μ-Biochar. Table S5. SEM-EDX for quantitative elemental analysis of surface of biochar flake, surface of μ-Biochar, and inner surface of μ-Biochar pore. Table S6. Average temperature and RH on the days of TGA. Table S7. Numerical data of moisture absorption test. Table S8. Full data of moisture absorption test. Table S9. Average compressive strength of biochar-containing cement composite.
