# Supplementary material for: Wood-Based Micro-Biochars in a Cement Mixture
Source: Molecules. 2025 Apr 24;30(9):1898. doi: 10.3390/molecules30091898 (PMC12073649; doi:10.3390/molecules30091898)
Supplement: Supplementary file 1 [file molecules-30-01898-s001.zip › molecules-3549849-supplementary.pdf]

Supporting Information

Wood-based Micro-Biochars in a Cement Mixture

Minkyeong Pyo [a,†], Jongsun Kim [b,†], Seungwook Seok [b],
Chan Ho Park [a,*], and Wonchang Choi [b,*]

[a] Department of Chemical, Biological, and Batterty Engineering, Gachon University, Seongnam, 13120, Republic of Korea

[b] Department of Architectural Engineering, Gachon University, Seongnam, 13120, Republic of Korea

† Equally contributed

* Correspondence: Corresponding author, E-mail: chhopark@gachon.ac.kr; wchoi@gachon.ac.kr;

Table of Contents

1. Supplementary Tables

- Average size of cells of biochar flake S1
- Average size of pores of μ-Biochar S2
- Particle size distribution results S3
- Pore characteristics analysis of μ-Biochar S4
- SEM-EDX for quantitative elemental analysis of biochar samples S5
- Average temperature and relative humidity on the days of TGA S6
- Numerical data of moisture absorption test S7
- Full data of moisture absorption test S8
- Average compressive strength of biochar-containing cement composite. S9

2. Supplementary Figures

- SEM image of cells which demonstrates honeycomb structure of biochar flake S1
- SEM image of pore of μ-Biochar S2
- Ratio of micropore, mesopore, and macropore of μ-Biochar S3
- SEM/EDX layered image and atom distribution of biochar samples S4
- Full plot of moisture absorption test S5
- SEM images of biochar with/without cement mortar S6

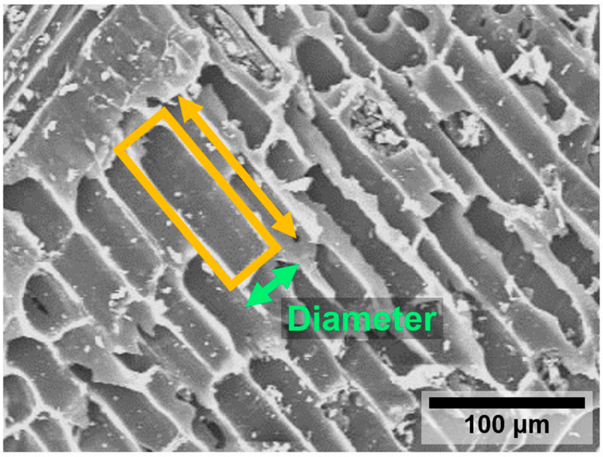

Figure S1. SEM image of cells which demonstrates honeycomb structure of biochar flake.

Table S1. Average size of cells of biochar flake.

Area (μm^2)	Diameter (µm)	Aspect ratio
418.6 ± 18.16	13.82 ± 0.327	2.286 ± 0.099

As the cells forming the honeycomb structure of biochar flakes are not uniform in shape, the area was calculated using the smallest rectangle enclosing each cell. The aspect ratio was determined using the long and short axes of rectangle, and diameter demonstrates the short side.

The micropores of biochar directly depend on the structural characteristics of its raw material, particularly its vessels. During pyrolysis, the shrinkage of wood causes the vessels and fibers to contract as well. In the study conducted by Rasa et al. [1], approximately 50% shrinkage was reported. Given that the average diameter of oak vessels is 26.77 µm [2], we can predict that the pore diameter of our biochar would be around 13.39 µm. This closely aligns with the measured value of 13.82 µm (Table S1).

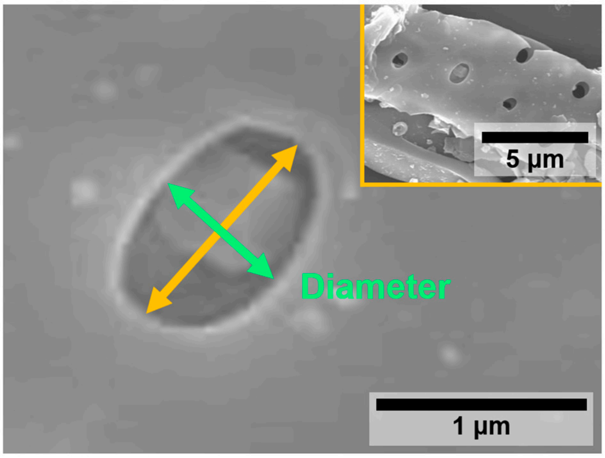

Figure S2. SEM image of pore of µ-Biochar.

Table S2. Average size of pores of µ-Biochar.

**Area (µm^2)**	**Diameter (µm)**	**Aspect ratio**
2.164 ± 0.23	1.373 ± 0.067	1.427 ± 0.043

Similarly, for the pores of u-Biochar, the area was calculated by determining the area of an ellipse using the long and short axes of the smallest rectangle enclosing the pore. The aspect ratio was calculated from these dimensions, and diameter demonstrates the short side.

Table S3. Particle size distribution results.

Description	Particle Size (μm)			
	D10	D50	D90	$D_{average}$
**1**	3.47	13.94	45.66	20.00
**2**	3.47	14.44	47.46	20.85
**3**	3.46	14.26	47.14	21.11
**Average**	3.47 ± 0.003	14.21 ± 0.15	46.64 ± 0.66	20.65 ± 0.34

Table S4. Pore characteristics analysis of μ-Biochar.

Method	Specific Surface Area (m^2/g)	Pore Volume (cm^3/g)	Pore Size (nm)
BET	244	0.12	1.98
BJH Adsorption	14.9	0.024	6.69
BJH Desorption	14.3	0.020	5.48

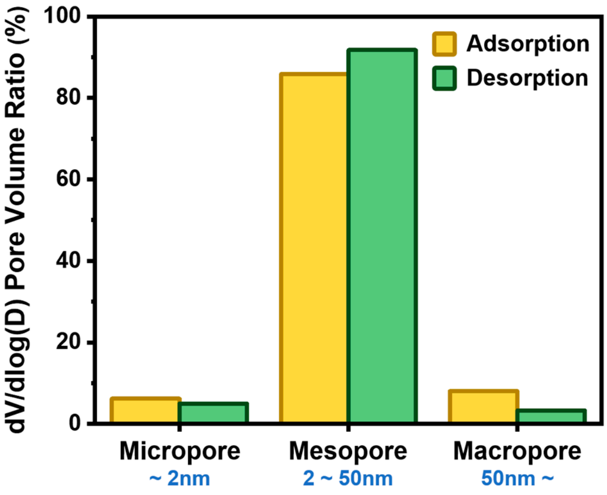

Figure S3. Ratio of micropore, mesopore, and macropore of μ-Biochar.

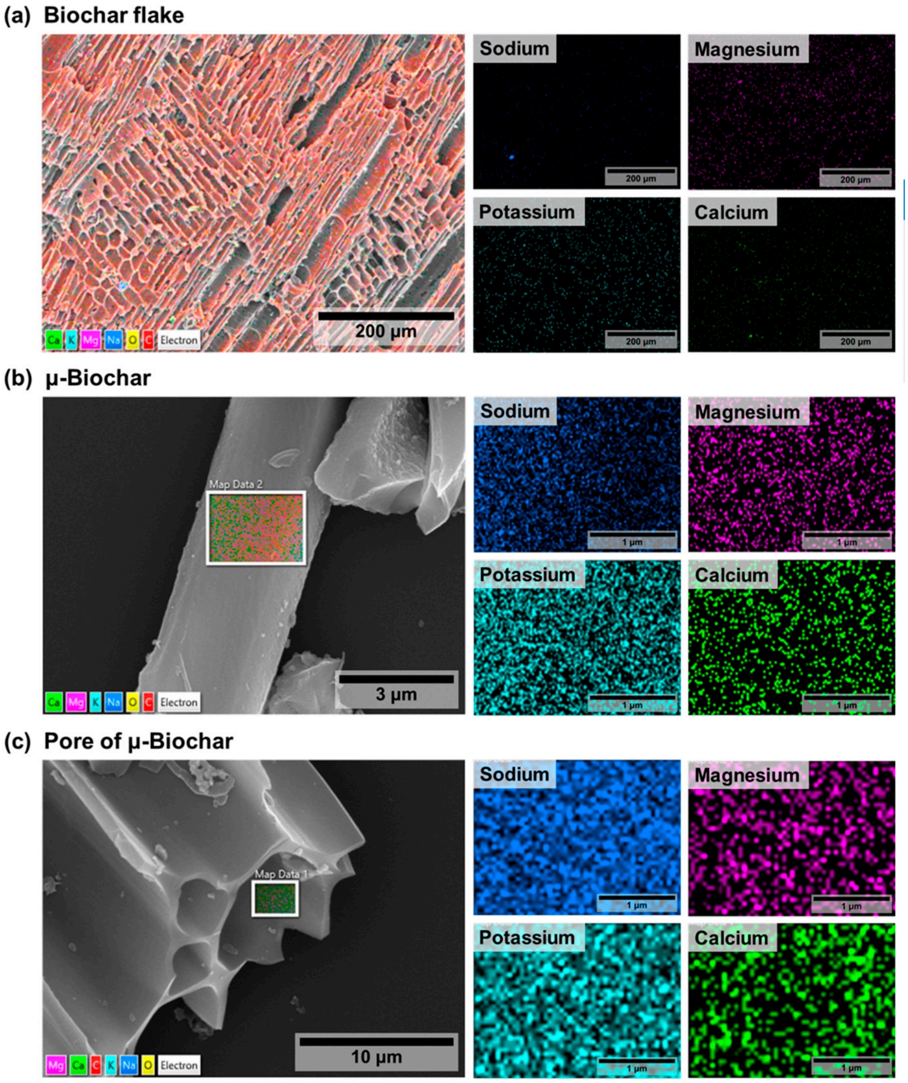

Figure S4. SEM/EDX layered image and atom distribution of sodium, magnesium, potassium, and calcium of (**a**) surface of biochar flake, (**b**) surface of μ-Biochar, and (**c**) inner surface of μ-Biochar pore.

Table S5. SEM-EDX for quantitative elemental analysis of surface of biochar flake, surface of μ-Biochar, and inner surface of μ-Biochar pore.

	Elemental Composition (wt%)					
	**Carbon**	**Oxygen**	**Sodium**	**Magnesium**	**Potassium**	**Calcium**
**Biochar flake**	93.70 ± 0.41	5.48 ± 0.39	0.09 ± 0.06	0.14 ± 0.06	0.25 ± 0.08	0.35 ± 0.08
**Surface of μ-Biochar**	61.21 ± 0.57	34.11 ± 0.55	2.13 ± 0.12	0.05 ± 0.06	2.33 ± 0.12	0.17 ± 0.08
**Inner surface of μ-Biochar**	30.57 ± 1.14	56.08 ± 1.08	7.93 ± 0.41	0.08 ± 0.31	5.01 ± 0.31	0.32 ± 0.22

Table S6. Average temperature and relative humidity on the days of TGA.

Day	March		April		August	
	Temperature (°C)	Relative Humidity (%)	Temperature (°C)	Relative Humidity (%)	Temperature (°C)	Relative Humidity (%)
**1**	-	-	12.2	32.0	-	-
**2**	-	-	15.4	37.5	29.5	79.4
**3**	-	-	-	-	30.2	75.0
**4**	-	-	-	-	31.8	69.3
**5**	-	-	-	-	30.0	75.5
**6**	-	-	-	-	29.5	74.8
**7**	-	-	-	-	29.6	72.0
**8**	-	-	-	-	28.5	77.5
**19**	6.7	60.8	-	-	-	-
**20**	4.4	46.5	-	-	-	-
**21**	5.0	46.5	-	-	-	-
**22**	10.4	57.0	-	-	-	-
**23**	13.0	66.0	-	-	-	-
**24**	16.2	42.5	-	-	-	-
**25**	10.6	63.0	-	-	-	-
**27**	10.4	60.4	-	-	-	-
**28**	10.3	81.8	-	-	-	-
**29**	7.5	73.0	-	-	-	-
**30**	8.8	56.5	-	-	-	-
**31**	10.6	39.9	-	-	-	-

Since the biochar have absorbed moisture over several days under ambient conditions, we calculated the average temperature and relative humidity over the period spanning from 7 days prior to the TGA measurement up until the day of analysis. The data collected in March and April were categorized as spring data, while those from August were designated as summer data in Gachon University, Korea.

Table S7. Numerical data of moisture absorption test.

Days	Biochar flake (wt%)	μ-Biochar (wt%)
**0**	0.00 ± 0.00	0.00 ± 0.00
**1**	8.25 ± 0.70	9.06 ± 0.71
**2**	8.58 ± 1.30	10.03 ± 0.73
**3**	8.89 ± 1.71	10.50 ± 0.70
**4**	9.19 ± 1.88	10.86 ± 0.63
**5**	9.67 ± 2.33	11.32 ± 0.79

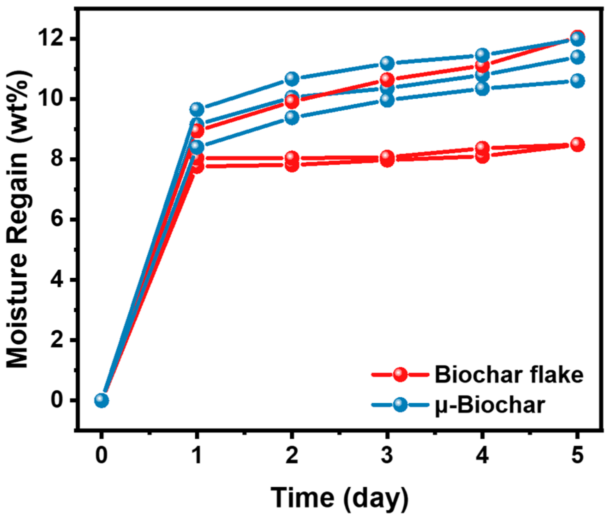

Figure S5. Plots of triplicated moisture absorption test.

Table S8. Full data of moisture absorption test.

Days	Batch 1		Batch 2		Batch 3	
	Biochar flake	μ-Biochar	Biochar flake	μ-Biochar	Biochar flake	μ-Biochar
**0**	0.000%	0.000%	0.000%	0.000%	0.000%	0.000%
**1**	7.765%	9.144%	8.939%	9.648%	8.068%	8.398%
**2**	7.813%	10.053%	9.912%	10.663%	8.068%	9.380%
**3**	7.974%	10.356%	10.636%	11.177%	8.069%	9.963%
**4**	8.102%	10.794%	11.107%	11.448%	8.361%	10.342%
**5**	8.483%	11.389%	12.050%	11.985%	8.490%	10.598%

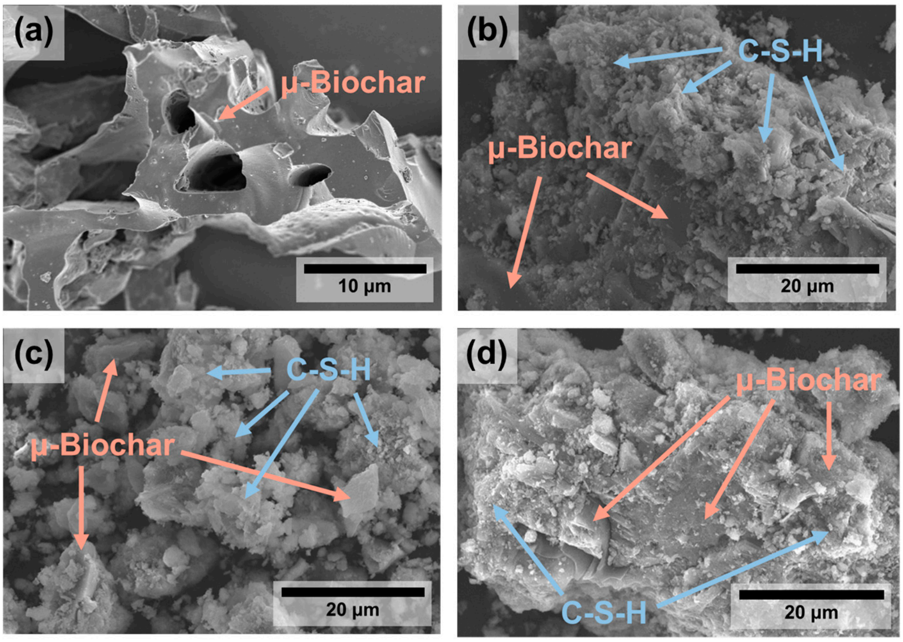

Figure S6. SEM images of (**a**) biochar without mixing with cement, and (**b**), (**c**), (**d**) mortar containing biochar (BC).

Table S9. Average compressive strength of biochar-containing cement composite.

Specimen	Compressive strength (MPa)	
	7 days	28 days
BC_0%	23.9 ± 1.53	29.6 ± 1.43
BC_1%	24.4 ± 1.02	30.2 ± 2.18
BC_3%	25.1 ± 0.72	30.9 ± 2.06
BC_5%	24.0 ± 1.62	30.4 ± 1.94

Reference

(1) Rasa, K.; Viherä-Aarnio, A.; Rytkönen, P.; Hyväluoma, J.; Kaseva, J.; Suhonen, H.; Jyske, T. Quantitative analysis of feedstock structural properties can help to produce willow biochar with homogenous pore system. *Ind. Crops Prod.* **2021**, *166*, 113475.
(2) Coomes, D. A.; Heathcote, S.; Godfrey, E. R.; Shepherd, J. J.; Sack, L. Scaling of xylem vessels and veins within the leaves of oak species. *Biol. Lett.* **2008**, *4* (3), 302-306.
